# How laws affect the perception of norms: Empirical evidence from the lockdown

**DOI:** 10.1371/journal.pone.0256624

**Published:** 2021-09-24

**Authors:** Roberto Galbiati, Emeric Henry, Nicolas Jacquemet, Max Lobeck

**Affiliations:** 1 Sciences Economiques Sciences Po, CNRS and CEPR, Paris, France; 2 Paris School of Economics and University Paris 1 Panthéon-Sorbonne, Centre d’Economie de la Sorbonne, Paris, France; University of Kwazulu-Natal, SOUTH AFRICA

## Abstract

Laws not only affect behavior due to changes in material payoffs, but they may also change the perception individuals have of social norms, either by shifting them directly or by providing information on these norms. Using detailed daily survey data and exploiting the introduction of lockdown measures in the UK in the context of the COVID-19 health crisis, we provide causal evidence that the law drastically changed the perception of the norms regarding social distancing behaviors. We show that this effect of laws on perceived norms is mostly driven by an informational channel and that the intervention made perceptions of social norms converge to the actual prevalent norm.

## Introduction

Individual behavior is affected both by material incentives, in particular those codified in laws and by social sanctions or rewards, which are embodied in behavioral norms. The interactions between laws and norms is of growing interest both in law and in economics. [1] propose a theoretical framework that formalizes two types of interactions. First, laws, by changing material payoffs, affect the behavioral norm understood as an equilibrium object: if fewer people take the condemned actions, the social stigma attached to these actions increases. Second, laws provide information on societal values, when there is an underlying uncertainty on the prevailing social norm. Both these mechanisms imply a shift in the perceived social norm as the result of the implementation of a law; because the norm did actually change in the first case, and through an informational channel in the second case. Considering such interactions between laws and norms is key to understanding why policies designed to foster cooperation fail or succeed (see [2, 3] for recent reviews).

In this paper, we take advantage of the lockdown measures introduced to face the COVID-19 pandemic and of rich survey data, gathered by [4] worldwide, to provide causal evidence that laws affect the perception of norms, and to disentangle the mechanisms behind the change. Our setting allows us to observe the effects of the law not only on perceived social norms (*i.e*., ones’ beliefs about what others consider appropriate) but also on personal norms (*i.e*., ones’ belief about what an appropriate behavior is). This allows us to build a measure of social norms that we can compare to perceived social norms. We focus on the case of Boris Johnson’s announcement of a nation-wide lockdown in the UK on the evening on March 23 2020. This setting has several key features that we can exploit to investigate the causal effect of laws on perceptions of the social norm. First, the implementation of this law came as a relative surprise. Indeed it represented a sharp change in the UK government’s strategy that has previously signaled strong reserves regarding total lockdowns. Second, the law was far reaching, including several different policies, such as lockdown and store closures, that had typically been more gradually implemented in other countries.

The law had an immediate impact on mobility. For example, the law reduced trips to parks by 30% within days. We provide evidence that this strong effect on behavior is supported by changes in the perceptions of the social norm. To that end, we examine how the law affected individual’s beliefs of whether *other* people believe that social-distancing measures are important. We test the causal effect of the lockdown announcement on the perception of social norms by comparing the daily responses of individuals that were interviewed before March 23 2020 in the UK with those that were interviewed after March 23 2020 in the UK. Trends in the perception of the social norm are controlled for by looking at the beliefs on the same days for respondents living in the set of control countries which did not impose a lockdown around March 23. The event study analysis coupled with a difference-in-differences estimation strategy show that the lockdown announcement significantly increased the likelihood that individuals believe that their compatriots positively view staying at home, closing stores, and not participating in social gatherings. The effect is very sizable. For example, it represents a 15 percentage points increase in the belief that other people think that stay at home measures should be followed. We show that these results are robust to various identification strategies. First, we vary the definition of the control group, excluding countries one at a time. Second, we restrict the control group to a set of countries that experienced no policy change during the sample period. Third, we show that our results hold when focusing solely on data observed on the days in the immediate neighborhood of March 23. Fourth, we exploit policy variations across all countries and show in a model with country and time fixed effects that the introduction of a lockdown increases the perceptions of the norm.

In the second part of the paper, we distinguish the two mechanisms proposed in [1] that can explain the shift in the perception of social norms. We study the impact of the law on personal norms, *i.e*., one’s own normative view about social distancing measures. First, we show that before the law was passed, there was a large gap between the social norm (measured as the weighted average personal norm) and the average perception of the social norm, a gap we call misperception. Second, by performing the same event study exercise as on the perceived social norm, we show that misperceptions sharply decrease after the implementation of the policy. This mostly reflects the fact that personal norms did not shift much. Overall, this suggests that the law mostly acted to provide information on the prevalent social norm, and helped to correct misperceptions about it.

This paper relates to different strands of literature. First, it contributes to a literature at the intersection of law and economics, that presents conceptual and theoretical mechanisms through which legal interventions interact with existing social norms. [5] is the first study to argue that, when people seek social approval, laws may provide information about prevailing social values. [6] study a context in which the effectiveness of laws depends on their coherence with social norms that drive social stigma for illegal behavior. [7, 8] take a different approach by studying the effects of laws on social norms in the context of evolutionary preferences adaptation. These papers examine the short and long-run effects of legal rules and show that they can have long-run effects on behavior because they affect the evolution of preferences. As previously mentioned, in [1] social norms directly affect the utility of individuals who care about their social image. Laws can, in this context, shift norms since certain behaviors become rarer, and thus more socially frowned upon, but can also bring information on the prevailing norm. Our work provides evidence consistent with the informational channel.

An experimental and empirical literature has examined how laws affect personal norms. A first wave of studies (*e.g*., [9], in a lab coordination game setting, [10–12], in the context of social dilemmas and [13], in the field) finds that laws affect behavior beyond changing monetary incentives. Most of these studies argue that the findings are consistent with the fact that laws shift personal norms. A second wave of studies provides direct evidence of this effect of laws. [14] show that the legal recognition of same sex unions is associated with a significant improvement in personal norms towards sexual minorities. Using a vignettes experiment, [15] show that laws exert an effect on whether people perceive certain behaviors as socially appropriate. However, very few papers examine how laws affect the perception of social norms, with the exception of [16, 17], that compare perceived social norms before and after the intervention in a single country—with no control of possible confounding effects of time varying factors.

Our work takes a comprehensive approach that goes beyond the scope of the aforementioned literature, since we study the impact of the law on the combination of personal and perceived norms and in particular study the impact on misperceptions. This enables us to disentangle the mechanisms proposed by [1].

Finally, our study also contributes to an emerging literature studying change in social norms in response to information provision. [18] show that, in Saudi Arabia, individuals misperceive the level of social support of women working outside of their homes and provide experimental evidence that correcting beliefs about others increases married men’s willingness to help their wives search for jobs. In a study focusing on the effect of information on the change of xenophobic social norms, [19] show that providing information about the constitutionality of a ban on Muslims holding public office affects people’s beliefs about the popularity of the policy. Our analysis shows that the introduction of new laws constitutes a different channel to provide information that corrects misperceptions about the prevailing social norms.

## Data

Our data come from a large online survey gathered by [4], henceforth FWH. The survey collects data on individual attitudes and beliefs concerning COVID-19 measures. The survey was launched on March 20 and we use responses up to March 30, resulting in over 99, 000 respondents from up to 58 countries. It is worth noting that the responses are not representative but representativity can be reconstructed using country-specific weights. We include all countries that had more than 200 respondents and completed the survey before March 30. We use the May 21 release of the data, which fixes some issues with the initial weight construction. The data contain daily information on respondents’ beliefs about four distinct social distancing measures: social gatherings, avoiding handshakes, closing stores and implementing a general curfew. For each of these measures, respondents were asked about their *perception of the social norm*, specifically whether they believe other individuals in their countries think that these measures should be adopted (see the supporting information S1 File, for detailed information about the variables and their definitions). We complement this information with external daily country-level data on coronavirus cases and deaths in the respondent’s country provided by John Hopkins University [20].

Boris Johnson’s March 23 announcement of a full and immediate lockdown in the UK is our main source of identification (the verbatim of the announcement is provided in the supporting information, S2 File). The lockdown prohibited citizens leaving their home except for one form of exercise per day, medical visits, shopping for basic necessities, and traveling to and from work if work from home is not possible. The lockdown also banned gatherings of more than 2 people and ordered non-essential shops to close. Importantly, this decision marked a stark change in the government’s approach to containing the epidemic. Indeed, as late as March 22 the prime minister recommended that individuals stay two meters apart when interacting outdoors, noting that he “want[s] people to be able to go to the parks and open spaces and to enjoy themselves—it is crucial for health and mental and physical wellbeing” [21]. The government in fact initially suggested that they would aim at achieving herd immunity. The measures also marked a change in policy relative to other countries that did not implement any further restrictions (e.g., Sweden) or that have already implemented such measures on a large scale (e.g. France, see [22]).

**Fig 1:**
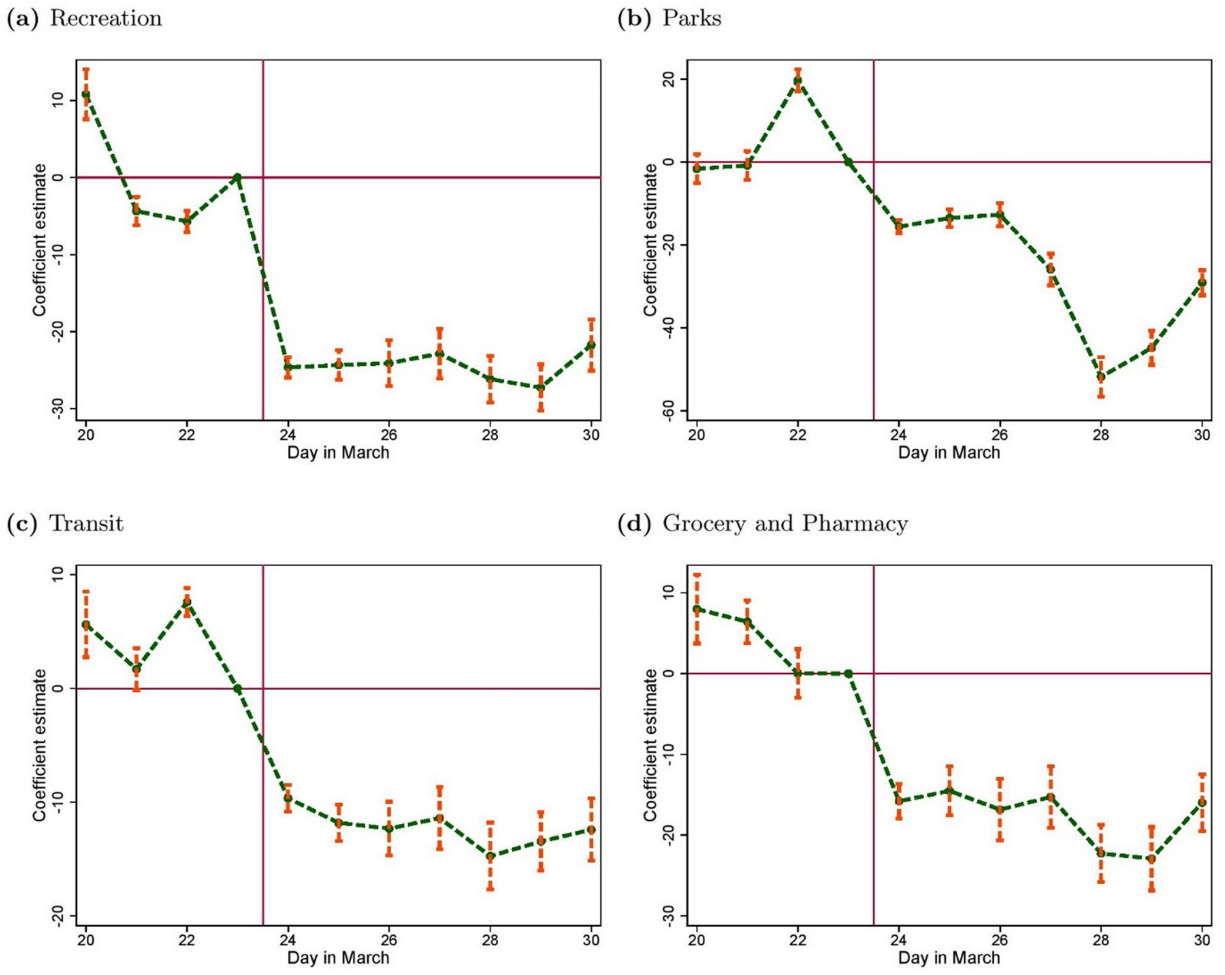
Mobility patterns in the UK. These figures plot the day-fixed effects of a specification that regresses a measure of mobility on days as well as Covid deaths and confirmed cases. The outcome variable is mobility data made publicly available by Google at https://www.google.com/covid19/mobility/. It characterizes in percent how many individuals spend time in (a) recreational areas, (b) parks, (c) transit, and (d) stores and pharmacy *relative* to a baseline. This baseline is the average number of visit on the same weekday between January 3rd and February 6th. A negative value thus means that there are fewer people at a given place than in the baseline period. The unit of observation is percent and the coefficients have to be interpreted as percentage points.

Still, it turned out that the enforcement was not as stringent as in other countries. Over the 3 months following the lockdown, less than 16, 000 fines for violation were issued (compared to more than a million in France), whereas the fine of 60 pounds was lower than the fines in many other countries (135 Euros in France). The police force had been instructed to favor discussion and education over sanctions. Nevertheless the law had a strong impact on the behavior of the population. Using mobility data made publicly available by Google and regressing different measures of mobility, controlling for the state of the pandemic in each country, we show in Fig 1 that all types of movements were significantly reduced after March 23rd in the UK, especially those for recreational purposes (these changes are all statistically significant, as shown in the supporting information, S3 Table). For example, the lockdown reduced time spent in parks by 29 percentage points compared to the average time spent in parks on the same weekday between January 3rd and February 6th. Note that this is the pure effect of the lockdown, controlling for mobility behavior earlier in the year before the lockdown.

**Fig 2:**
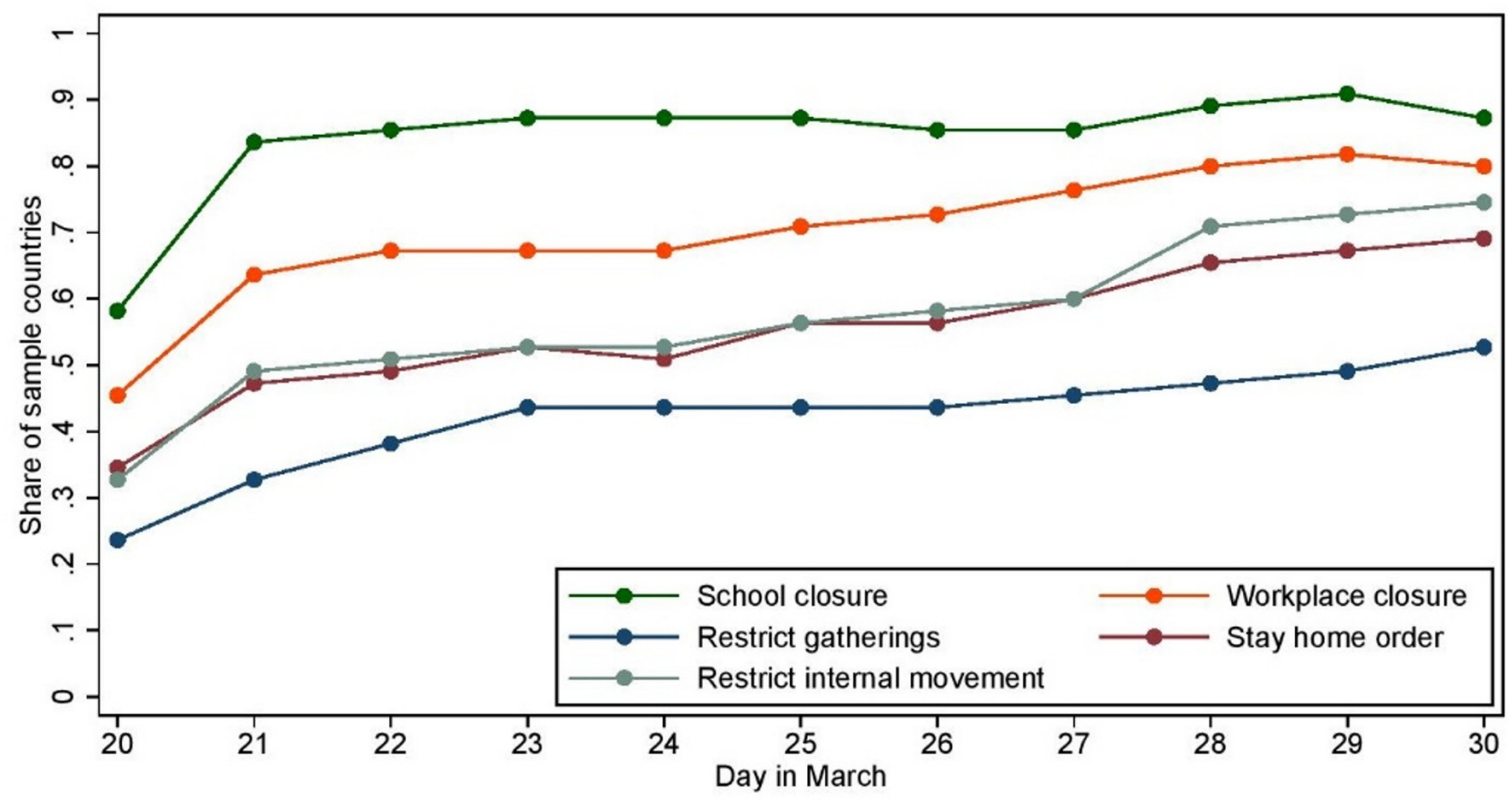
Timeline of the policies implemented in the control group. The figure reports the fraction of countries in the control group where the corresponding policy was enforced on each day provided on the x-axis.

In what follows we will examine whether this large impact of the law on mobility is correlated with changes in the perception of the social norm (it is worth noting that our data does not allow us to directly measure the relationship between mobility and norms; in the supporting information, S4 Table, we report tentative results based on questions in the survey about the intention to stay at home). To that end, we exploit the lag in the implementation of similar policies in other countries. Based on the information provided by the Oxford COVID-19 Government Response Tracker (that tracks social distancing policies on the country level by day [22]), Fig 2 displays the timeline of the implementation of several policies (school closings, workplace closing, cancellation of public events, stay-at-home requirements, closure of public transportation, restrictions on internal movements and restrictions on international travel) in all countries available in the survey (we focus on the ‘containment and closure’ category and restrict our study to only mandatory policies, disregarding simple recommendations from the governments). Even though there was a gradual adoption of more stringent policies around the world, none of these countries experienced a change as drastic (all policies being implemented on the same time) and sudden (hence unexpected) as the one observed in the UK. Our identification strategy leverages this sharp discontinuity that happened on March 23rd in the UK. In our main specification of interest, we use all countries on which data is available as a control for changes in norms over time. This implies that our control group will include countries in which some policy change occurred during the sample period, although it is unlikely that these small and well-anticipated policy changes came with a change in norms (our robustness checks, presented in Section Robustness analysis, aim to ascertain that our results are not driven by such changes). This choice aims to preserve the generality of our results, as focusing on the subset of countries in which no change occurs raises the risk of conditioning the results on a selected set of countries whose policy response differs from the rest of the world.

## Results

### The causal effect of the law on perceived social norms

**Fig 3:**
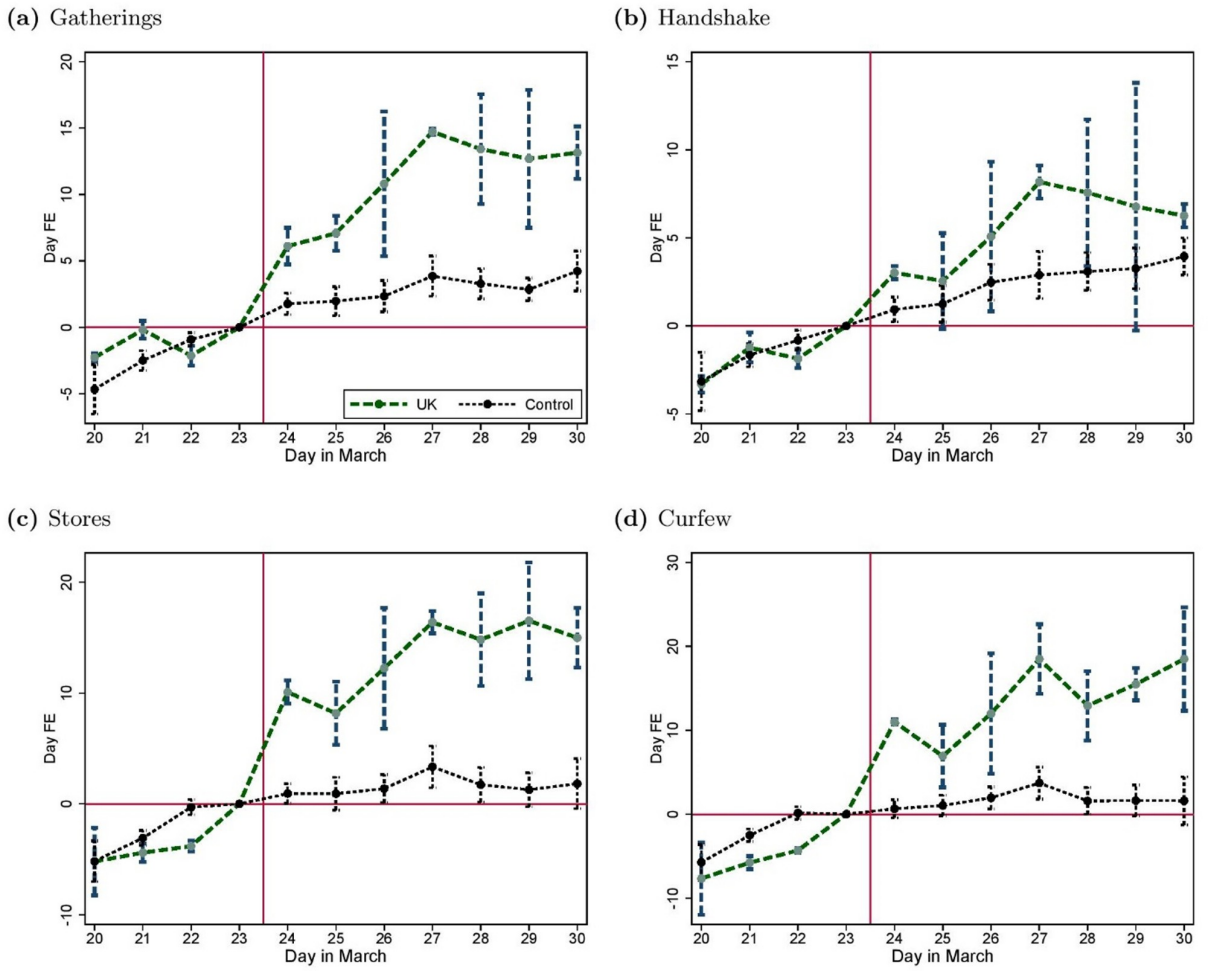
Time pattern of perceived social norms in the UK and the control group. The figure reports the day-fixed effects along with their 95% confidence intervals from separate regressions in the UK and in the control group of the perceived social norm measured in the survey about (a) social gatherings, (b) handshaking, (c) stores closure and (d) a total curfew, controlling for country, age, gender, education, and income-fixed effects, as well as household composition. Standard errors are clustered at the country-gender level.

Fig 3 provides descriptive support for our main result. For each of the four policy measures (forbidding social gatherings, prohibiting handshake, closing stores and introducing a curfew), we report the day-fixed effects for the regressions of the perceived social norm in the UK and in the control group countries. We control for differences in observed heterogeneity by including country, age (measured in bins of 5 years), gender, education, income-fixed effects and a measure of household composition. For all four social distancing measures the pre-trends are similar prior to the announcement by Boris Johnson. However there is a sharp discontinuity in the trend for the UK after March 23rd.

We statistically test for these effects by running difference-in-difference (DiD) regressions on the perceived social norm of each of the four policies, measured at the individual level, *i*, on each day *d*. We generalize the approach compared to the usual linear model by introducing day and country fixed-effects (which allow for a non-linear effect of the post-treatment and the treatment-group dummy variables) and estimate the following model (bold letters denote column vectors):

$$\begin{array}{c} y_{id}=\alpha +\beta Post_{d}\times UK_{i}+{Country}_{i}^{\prime }\gamma +{Day}_{d}^{\prime }\phi +x_{id}^{\prime }\kappa +u_{id},\;\forall i,d \end{array}$$ (1)

The outcome variable, *y*_*id*_, denotes either one of the four perceived social norms, or one of the four measures of misperceptions as defined in Section Robustness analysis. The control variables in vector ***x*** notably include (current and lagged) measures of country-date variations in COVID-19 deaths and confirmed cases. We also include country-age-gender, country-education, income quintile, and day fixed-effects as well as a control for household composition. Last, we account for individual correlation in unobserved heterogeneity both over time within countries and between individuals within countries by clustering the standard errors at the country-gender level. In the main tables, we only report the estimates of the *β* parameters. The full results tables are provided in the supporting information, S5 Table. Also note that we do not control for normative beliefs in our preferred regressions on perceived social norms, since they are likely to be endogenous to unobserved heterogeneity generating differences in perceived social norms. The robustness check available from the authors upon request, shows that the results still holds when normative beliefs are controlled for and, if anything, are stronger both statistically and in terms of magnitude.

The results of the DiD estimates are provided in the first row of Table 1, where we perform the estimation both on the entire set of countries available in the sample (panel A) and on the more homogeneous subset of Western and Northern European countries (panel B; the list is provided in the supporting information, S1 Table). The lockdown announcement has a strong positive effect on the perceived social norm, for all of the different social distancing measures (note that their unit are the same). The effect is the strongest for the implementation of a general curfew and the closure of stores. It is weaker for social gatherings and positive, but small, for the no handshake policy. The effect is similar in magnitude if we only compare the UK respondents to respondents from other North-Western countries (panel B).

**Table 1:** DiD estimates of the effect of the UK lockdown.

	Gatherings	Handshake	Stores	Curfew	*N*	Clusters
**A. Full sample**						
**Perceived norm**	7.370*** (1.287)	3.133** (1.158)	12.907*** (1.386)	13.950*** (1.311)	94,544	155
**Misperceptions**	-6.182*** (1.518)	-0.278 (1.499)	-6.037*** (1.649)	-3.910* (1.630)	91,182	137
**B. Western and Northern Europe**						
**Perceived norm**	7.825*** (1.429)	4.262*** (1.185)	14.284*** (1.913)	14.405*** (1.764)	37,745	38
**Misperceptions**	-7.622*** (2.020)	-1.699 (1.153)	-6.773* (2.979)	-4.269* (1.958)	37,745	38

**Note**. This table presents the difference-in-difference estimates (SE) (Post × UK variable) from regressions of perceived social norms (first row in each panel) and misperceptions (second row) on country-age-gender, country-education, income quintile, and day fixed-effects as well as controls for household composition and COVID-19 statistics (lagged and current confirmed cases and deaths). The sample uses all countries and respondents that answered between March 20 and 30. The second line only uses countries for which we have enough responses to compute a misperception variable. Standard errors are reported in parentheses and clustered at the country-gender level.

Significance levels:

*5%,

**1%,

***0.1%.

#### Robustness analysis

Our identification strategy relies on the sudden and unexpected change in law that occurred in the UK, to measure the causal effect of this change on the perception of norms. It is thus conditional on the assumption that *(i)* the announcement is the only reason why norms change in the UK; *(ii)* countries in the control group provide a counterfactual and did not experience similar shocks affecting the norms. In the following, we consider several alternative identification strategies, aimed at assessing the robustness of our results to the relaxation of these assumptions. The main results are presented in Table 2.

**Table 2:** Robustness checks.

	Gatherings	Handshake	Stores	Curfew	*N*	Clusters
**A. March 22 vs March 24**						
**Full Sample**	5.420*** (0.821)	2.819*** (0.709)	11.967*** (1.420)	12.446*** (1.501)	47,366	142
**Western and Northern Europe**	4.798*** (0.824)	2.809*** (0.577)	11.293*** (1.682)	11.600*** (1.453)	20,029	37
**B. Effect of a strict lockdown on pooled data**						
**Full Sample**	2.721*** (0.647)	0.852 (0.591)	5.558*** (0.968)	7.193*** (1.006)	94,544	155
**Western and Northern Europe**	3.303*** (0.401)	2.058*** (0.420)	7.394*** (0.801)	8.146*** (0.815)	37,745	38
**C. Countries with no change in their lockdown policy**						
**Full Sample**	8.043*** (1.188)	3.392** (1.113)	14.051*** (1.299)	14.886*** (1.279)	66,337	103
**Western and Northern Europe**	8.367*** (1.168)	4.454*** (0.938)	14.899*** (1.422)	14.864*** (1.206)	28,172	29

**Note**. Standard errors are reported in parentheses and clustered at the country-gender level. The full results are available from the authors upon request.

Significance levels:

*5%,

**1%,

***0.1%.

We address the first concern in two ways. First, as explained in Section Data, our main source of identification is the discontinuity in the government’s policies in the UK on March 23. As an alternative to the difference-in-difference estimates using the entire sample period, we narrow the analysis to the neighborhood of the discontinuity and focus solely on data observed on March 22 and March 24 (see panel A). The results confirm that the March 23 announcement is the main source of variations in norms observed in our main specification. It could still be the case, however, that the results are driven by other events that occurred in the UK on that same day. To address this concern, we exploit changes in the timing of the introduction of lockdowns in all countries over the time period of interest (lockdowns were introduced in 49 out of the 172 countries represented in our sample), by regressing perceived social norms on country fixed effects, day fixed effects and an indicator variable measuring whether a lockdown is in place (see panel B). The results on pooled data show that the introduction of a lockdown is correlated with a significant and large increase in perceived social norms. While these results are useful to confirm that the change observed in the UK is unlikely to be due to simultaneity with unrelated events, it is worth stressing that the UK provides a unique situation of a sudden and unanticipated lockdown, hence achieving a more convincing and more conservative identification of its effect.

The second concern is that our estimates could capture the announcement of similar policies in the countries in the control group. Fig 2 indeed shows that some policy variation does occur in the control group during the sample period—although the trend is very flat around the discontinuity. To eliminate these variations from the estimated effect, we run the difference-in-difference estimation in (1) on the control group composed only of countries in which no policy variation occurs between March 20 and March 30 (see panel C). This exercise leads to very similar estimates in terms both of magnitude and statistical significance. To further investigate this issue, our last robustness checks rely on variations in the composition of the control group used to generate the estimates provided in Table 1. We replicate the estimation based on the same control group, but we remove each country one at a time (the results are provided in the supporting information, S1 Fig). All estimated coefficients are statistically indistinguishable from the baseline coefficient (first line in the figure). This shows that our results are not driven by the dynamics in one particular country.

### The gap between perceived and actual norm

The results in the previous section clearly show that the law had a strong effect on individual perceptions of the social norm. We now examine the mechanism that could lie behind this effect. [1] suggest two main channels; the first is that, by changing the material payoffs of the socially desired action, the law also affects the norm of behavior resulting from equilibrium behavior. In particular, if individuals start adopting the prescribed action because of the sanctions imposed by laws, those who still do not take this action will signal their extreme attitudes and carry worse stigma. Here, the law thus directly affects the social norm. A second channel is that the law may bring information on the social norm, even if the norm itself remains unchanged.

These two explanations notably differ regarding whether the social norm actually changes. We build a measure of actual social norms using an additional variable in the survey asking respondents about their *personal norm*, *i.e*. whether they think people should comply with the policy (the variables are described in the supporting information, S1 File). If the information about the prevalent social norm was perfect, the average over the entire population of the personal norms should be equal to the average perception of the social norm. We thus build a measure of the actual social norm based on the average personal norm. To account for the fact that the sample is not representative of the entire population, we compute the weighted average of this variable at the country level separately before and after March 23, where the weights rescale the data to make our sample representative of the gender-age-income-household size composition of the population at the country level.

**Fig 4:**
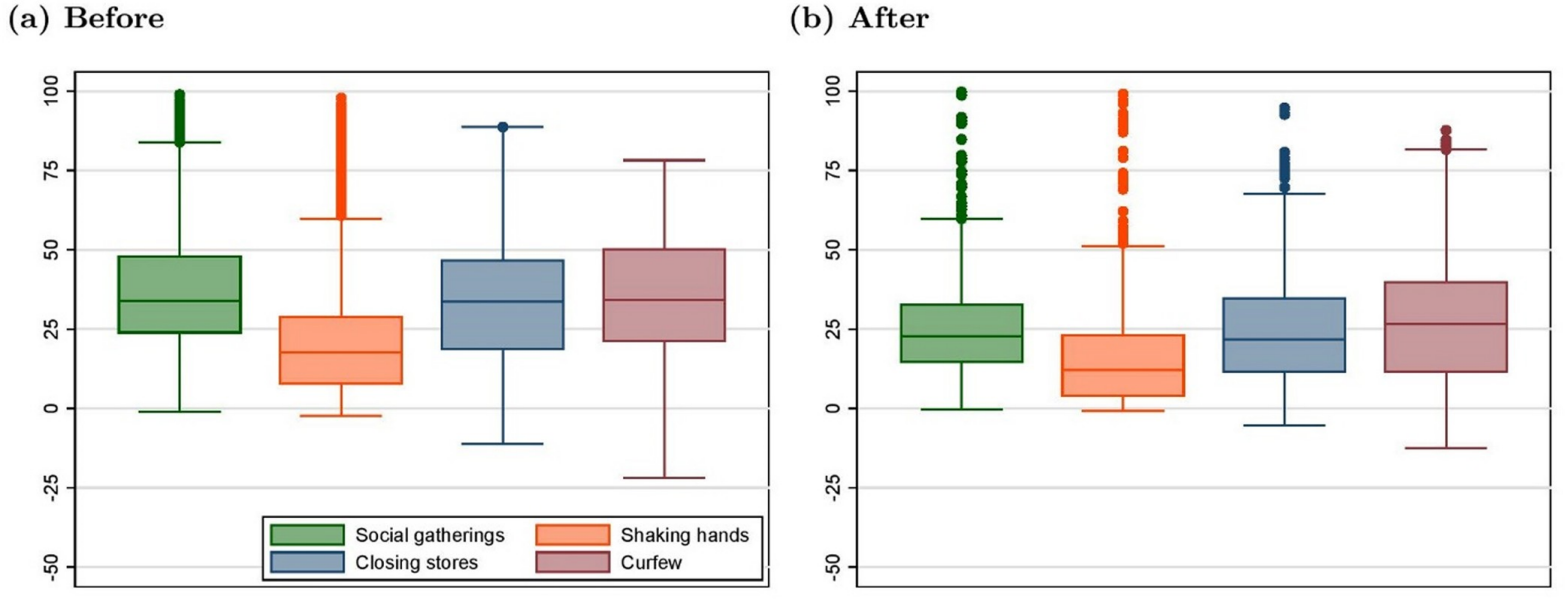
Before-after comparison of misperceptions in the UK. Discrepancy between the norm and the individual perception of the norm in the UK before and after March 23. The horizontal line in the middle of the box characterizes the median. The upper (lower) ends of the box characterizes the 75th (25th) percentile. The upper (lower) ends of the vertical lines are the upper (lower) adjacent values. Points above (below) are outliers.

Using this measure, we first show that there was an initial gap between the actual social norm and the average perception of the social norm in the UK, a gap we call misperceptions. Fig 4 presents misperceptions before March 23 in the left panel and after in the right panel. The left panel clearly shows that before the lockdown, misperceptions were very high. In other words, the perceptions of the social norm were significantly below the norm itself for most dimensions, except for handshake where the discrepancy was much lower. The right panel shows that after March 23, this misperception sharply drops.

**Fig 5:**
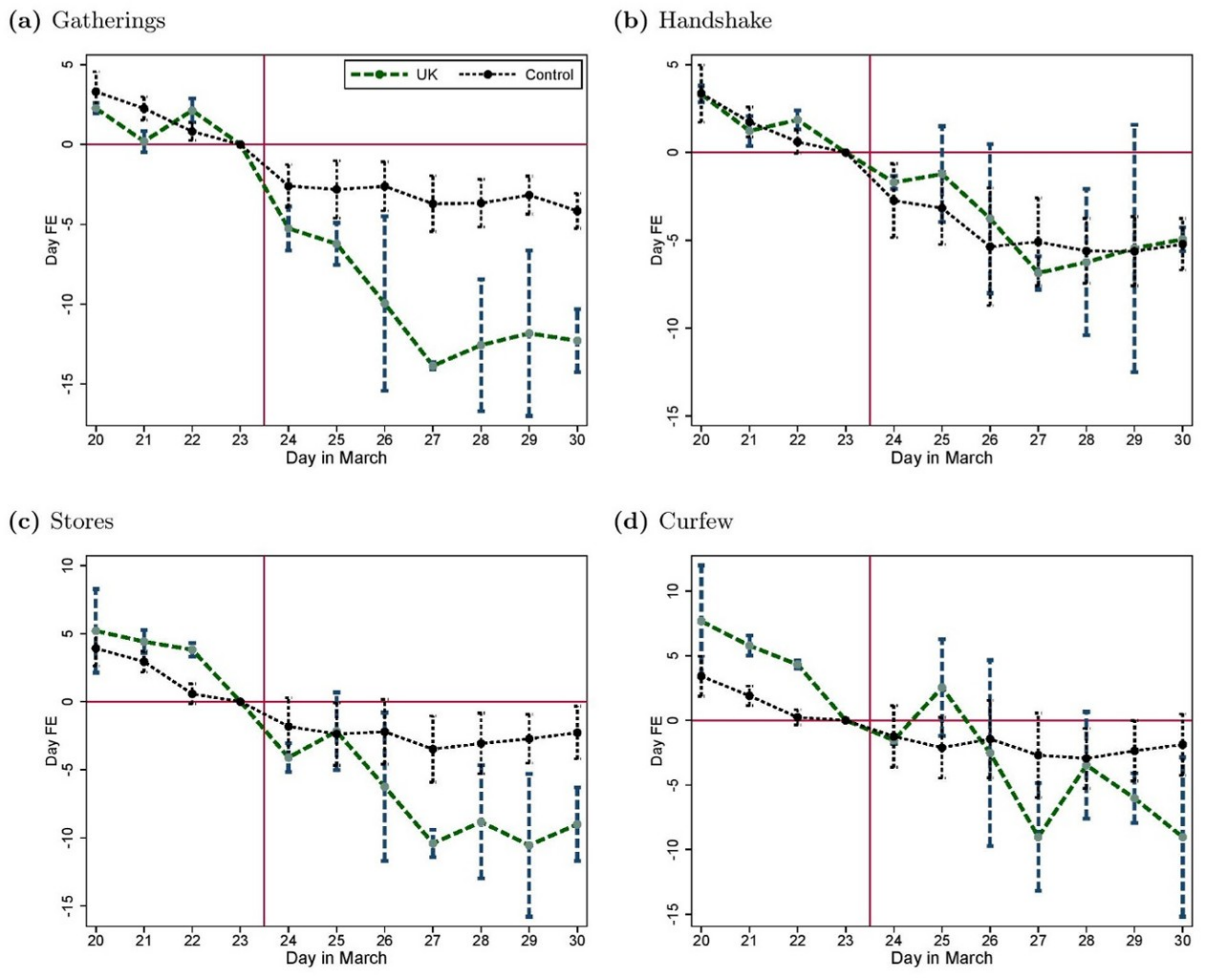
Time pattern of the misperceptions in the UK and the control group. The figure reports the day-fixed effects along with their 95% confidence intervals from separate regressions in the UK and in the control group of the misperception about (a) social gatherings, (b) handshaking, (c) stores closure and (d) a total curfew, controlling for country, age, gender, education, and income-fixed effects, as well as household composition. Standard errors are clustered at the country-gender level.

This evolution observed in the raw data is confirmed in Fig 5 where we replicate Fig 3 but for the misperception measure. As shown in Table 1 (second row in each subpanel), these results all are statistically significant based on the DiD estimates from linear models similar to (1) in which the misperception is used as a dependent variable. This effect on misperceptions, which echoes the effect on the perceived social norms, is essentially driven by the fact that personal norms themselves do not change (see the supporting information, S2 Fig). We also ascertain that the effect is not driven by the use of weights thanks to a replication of the DiD estimation of the effect on perceived social norms using weighted data (see the supporting information, S7 Table). This set of results suggests that the most plausible channel is that the law changed the perceptions of the social norm without actually changing the norm itself. In fact, for handshakes, the dimension where misperceptions was initially the lowest, the law had virtually no effect on the perceptions of the social norm. This interpretation is further substantiated by the analysis of the heterogeneity of the response of perceived social norms to the UK lockdown. When interacting the treatment effect with subjective perception variables in separate models, the only dimension of heterogeneity that seems to matter is how well individuals are informed about COVID-19 itself, measured as the gap between their estimate of the number of COVID-19 cases and the actual number in their country. The higher that gap, the more the perception of the social norm relative to the curfew variable increases. These results, along with the results from interactions with individual covariates, are provided in the supporting information S8 and S9 Tables, which show that our results remain stable when controlling for these interactions.

## Remarks and conclusions

In this paper, we use the announcement of a sudden lockdown in the UK to provide causal evidence that laws affect the perception of social norms in the population. We also show that the most plausible mechanism is an informational channel rather than a direct change in the social norms. The UK context had several features that favoured this informational channel. First, the population was initially pessimistic about the prevalent norm, as indicated by the large misperception gap before March 23rd, leaving room for new information to affect beliefs. Second, as described in Section Data, the enforcement of the law was weaker in the UK than in other countries, decreasing the potential for a direct impact on social norms themselves. In this context, our results show that the law provides information, resulting in a large change in the perceptions of social norms. Our study thus sheds new light on the mechanisms explaining why and under what circumstances laws are effective, and contributes to a better understanding of how perceptions about prevailing social norms are formed.

In conclusion, it is worth discussing to what extent the particular context we study differs compared to other environments where laws may affect social norms and their perception. An important difference relates to the timing of the implementation of the new legal rule and to the procedure of its adoption. Laws are usually debated in parliaments and subject to public discussion (for example, this has occurred in the case of the introduction of same-sex marriage), while the UK lockdown was a sudden decision of the government. This specific setting has clear advantages in terms of identification since it relies on a sudden change. Moreover, even though the policy is not publicly debated, it still provides information about the underlying norm. Indeed, in a democracy, accountability constrains politicians to consider voters’ preferences. Thus, British citizens, who are aware of this accountability mechanism, receive a clear message regarding the perception the government has of the general support in the population for this measure.

## Supporting information

S1 File Variables and data source. Document illstrustrating variable and weight construction.
(PDF)

S2 File Verbatim of Boris Johnson’s March 23 announcement. (PDF)

S3 File Note on S8 and S9 Tables. (PDF)

S1 Fig Step deletion of countries from the control group. The figure reports the point estimates along with 95% confidence intervals resulting from the difference-in-difference estimation in (1) performed on control groups resulting from the step deletion of each country one after the other. The countries iso-code are defined in S1 Table.
(TIF)

S2 Fig Time-pattern of personal norms in the UK and the control group. For each of the four policy measures, the figure reports the day-fixed effects from individual personal norms in the UK and in control group countries, controlling for country-, age-, gender-, education-, and income-fixed effects, as as well as a measure of household composition. The results from difference-in-difference estimates of the effect of March 23 announcement on individual personal norms, available from the authors upon request, confirm that the announcement has a non-significant effect regarding both handshaking and social gatherings, and a small but statistically significant effect regarding stores closure and a total curfew.
(TIF)

S1 Table Variables and data source. Table reporting the distribution of respondents across countries.
(PDF)

S2 Table Variables and data source. Table providing a comparison of the descriptive statistics observed in the UK and in other countries.
(PDF)

S3 Table Effect of March 23 lockdown measures on movements in the UK. This Table reports the results of the Difference-in-Difference estimation (*Post × UK* variable) of the March 23 lockdown decision in the UK on mobility data collected from Google’s publicly available community mobility reports. Each outcome variable is the change in spending time at a given place relative to the median value of the same weekday in the January 3-February 6 period. The specification controls for COVID-19 statistics (lagged and current confirmed cases and deaths) as well as a non-linear effect of the group and Post variable through day- and country fixed-effects.
(PDF)

S4 Table Relationship between self-reported mobility and norms. (PDF)

S5 Table Full results from Table 1 (norm as outcome variable). (PDF)

S6 Table Full results from Table 1 (misperception of norm as outcome variable). (PDF)

S7 Table Replication of the results on weighted data. This table provides the DiD estimate in (1) using weights. The weights provide representativity on the country level by age, income quintile, and education.
(PDF)

S8 Table Heterogeneity of the DiD estimate according to individual covariates. (PDF)

S9 Table Heterogeneity of the DiD estimate according to subjective perception variables. (PDF)
