# Supplementary material for: How laws affect the perception of norms: Empirical evidence from the lockdown
Source: PLoS One. 2021 Sep 24;16(9):e0256624. doi: 10.1371/journal.pone.0256624 (PMC8462721; doi:10.1371/journal.pone.0256624)
Supplement: S1 File — Document illstrustrating variable and weight construction. (PDF) [file pone.0256624.s001.pdf]

The entire set of variables available in the data is described in FWH. We only describe below the variables used as the main outcomes in the analysis.

Perceived social norms. Our measure of perceived social norms come from questions about the perception of others' beliefs about Coronavirus measures:

- *sob_social*: How many of 100 people in your country do you think believe that participation at social gatherings should be cancelled because of the coronavirus right now? [slider ranging from 0 to 100 — initiated at 0]
- *sob_handshake*: How many of 100 people in your country do you think believe that one should not shake other people's hands because of the coronavirus right now? [slider ranging from 0 to 100 — initiated at 0]
- *sob_stores*: How many of 100 people in your country do you think believe that all shops in your country other than particularly important ones, such as supermarkets, pharmacies, post offices, and gas stations, should be closed because of the coronavirus right now? [slider ranging from 0 to 100 — initiated at 0]
- *sob_curfew*: How many of 100 people in your country do you think believe there should be a general curfew in your country (with the exception of grocery shopping, necessary family trips, and the commute to work) because of the coronavirus right now? [slider ranging from 0 to 100 — initiated at 0]

Misperception. To build a measure of misperception, we rely on questions about personal norms:

- *fob_social*: "What do you think: should people in your country cancel their participation at social gatherings because of the coronavirus right now?" [No = 0; Yes = 1]
- *fob_handshake*: "What do you think: should people in your country not shake other people's hands because of the coronavirus right now?" [No = 0; Yes = 1]
- *fob_stores*: "What do you think: should all shops in your country other than particularly important ones, such as supermarkets, pharmacies, post offices, and gas stations, be closed because of the coronavirus right now?" [No = 0; Yes = 1]

- *fob_curfew:* “What do you think: should there be a general curfew in your country (with the exception of grocery shopping, necessary family trips, and the commute to work) because of the coronavirus right now?” [No = 0; Yes = 1]

For each policy, we compute the weighted average of this variable at the country level, separately before and after March 23. The weights aim at restoring the national representativity of the sample regarding observed heterogeneity available in the data. However, for some countries the dataset did not include the necessary information to construct weights. Thus, the total sample shrinks from 94,544 to 91,182 for models that study misperception as an outcome. Note that the European sample is not affected by this.

Weights. The dataset also contains census information about the distribution of the population in each country over age, gender, education, income and household composition (assuming independence in the population between these characteristics). We use this additional source of information to build weights rescaling the observations available in the sample to make them representative of the country population.
