# Supplementary material for: How laws affect the perception of norms: Empirical evidence from the lockdown
Source: PLoS One. 2021 Sep 24;16(9):e0256624. doi: 10.1371/journal.pone.0256624 (PMC8462721; doi:10.1371/journal.pone.0256624)
Supplement: S2 File — (PDF) [file pone.0256624.s002.pdf]

According to UK government official communication, the speech reads as follows (`https://www.gov.uk/government/speeches/pm-address-to-the-nation-on-coronavirus-23-march-2020`, text emphasized by us):

Good Evening,

The coronavirus is the biggest threat this country has faced for decades – and this country is not alone.

All over the world we are seeing the devastating impact of this invisible killer.

And so tonight I want to update you on the latest steps we are taking to fight the disease and what you can do to help.

And I want to begin by reminding you why the UK has been taking the approach that we have.

Without a huge national effort to halt the growth of this virus, there will come a moment when no health service in the world could possibly cope; because there won't be enough ventilators, enough intensive care beds, enough doctors and nurses.

And as we have seen elsewhere, in other countries that also have fantastic health care systems, that is the moment of real danger.

To put it simply, if too many people become seriously unwell at one time, the NHS will be unable to handle it - meaning more people are likely to die, not just from Coronavirus but from other illnesses as well.

So it's vital to slow the spread of the disease.

Because that is the way we reduce the number of people needing hospital treatment at any one time, so we can protect the NHS's ability to cope - and save more lives.

And that's why we have been asking people to stay at home during this pandemic.

And though huge numbers are complying - and I thank you all - the time has now come for us all to do more.

From this evening I must give the British people a very simple instruction - you must stay at home.

Because the critical thing we must do is stop the disease spreading between households.

That is why people will only be allowed to leave their home for the following very limited purposes:

- ***shopping for basic necessities, as infrequently as possible***

- ***one form of exercise a day - for example a run, walk, or cycle - alone or with members of your household;***
- ***any medical need, to provide care or to help a vulnerable person; and travelling to and from work, but only where this is absolutely necessary and cannot be done from home.***

That's all - these are the only reasons you should leave your home.

You should not be meeting friends. If your friends ask you to meet, you should say No.

You should not be meeting family members who do not live in your home.

You should not be going shopping except for essentials like food and medicine - and you should do this as little as you can. And use food delivery services where you can.

If you don't follow the rules the police will have the powers to enforce them, including through fines and dispersing gatherings.

To ensure compliance with the Government's instruction to stay at home, we will immediately:

- ***close all shops selling non-essential goods, including clothing and electronic stores and other premises including libraries, playgrounds and outdoor gyms, and places of worship;***
- ***we will stop all gatherings of more than two people in public – excluding people you live with;***
- ***and we'll stop all social events, including weddings, baptisms and other ceremonies, but excluding funerals.***

Parks will remain open for exercise but gatherings will be dispersed.

No Prime Minister wants to enact measures like this.

I know the damage that this disruption is doing and will do to people's lives, to their businesses and to their jobs.

And that's why we have produced a huge and unprecedented programme of support both for workers and for business.

And I can assure you that we will keep these restrictions under constant review. We will look again in three weeks, and relax them if the evidence shows we are able to.

But at present there are just no easy options. The way ahead is hard, and it is still true that many lives will sadly be lost.

And yet it is also true that there is a clear way through.

Day by day we are strengthening our amazing NHS with 7500 former clinicians now coming back to the service.

With the time you buy - by simply staying at home - we are increasing our stocks of equipment.

We are accelerating our search for treatments.

We are pioneering work on a vaccine.

And we are buying millions of testing kits that will enable us to turn the tide on this invisible killer.

I want to thank everyone who is working flat out to beat the virus.

Everyone from the supermarket staff to the transport workers to the carers to the nurses and doctors on the frontline.

But in this fight we can be in no doubt that each and every one of us is directly enlisted.

Each and every one of us is now obliged to join together.

To halt the spread of this disease.

To protect our NHS and to save many many thousands of lives.

And I know that as they have in the past so many times.

The people of this country will rise to that challenge.

And we will come through it stronger than ever.

We will beat the coronavirus and we will beat it together.

And therefore I urge you at this moment of national emergency to stay at home, protect our NHS and save lives.

Thank you.
