# Supplementary material for: How laws affect the perception of norms: Empirical evidence from the lockdown
Source: PLoS One. 2021 Sep 24;16(9):e0256624. doi: 10.1371/journal.pone.0256624 (PMC8462721; doi:10.1371/journal.pone.0256624)
Supplement: S3 File — (PDF) [file pone.0256624.s003.pdf]

Tables S8 and S9 below provide the results from separate regressions in which we allow the DiD estimate in (1) to interact with observed individual covariates (S8 Table) and subjective variables (S7 Table). For each set of regressions, we report the coefficient of the corresponding variable, the resulting DiD estimate ($Post \times UK$ variable) as well as the interaction. The subjective variables come from the "Perceptions of government/public response and efficacy" part of the survey, and are measured as:

- *perceivedeffectivnes*: What do you think: How effective are social distancing measures (e.g., through a general curfew) to slow down the spread of the coronavirus? [5- point scale; 1 = Not at all effective; 2 = Not effective; 3 = Neither effective nor ineffective; 4 = Effective; 5 = Very effective]

- *govtrust*: How much do you trust your country's government to take care of its citizens? [5-point scale; 1 = Strongly distrust; 2 = Somewhat distrust; 3 = Neither trust nor distrust; 4 = Somewhat trust; 5 = Strongly trust]

- *govfact*: How factually truthful do you think your country's government has been about the coronavirus outbreak? [5-point scale; 1 = Very untruthful; 2 = Somewhat untruthful; 3 = Neither truthful nor untruthful; 4 = Somewhat truthful; 5 = Very truthful]

For each variable, we generate dummy variables equal to 1 if the individual's response is above the midpoint of the Likert scale, and 0 if it is below.
