# Supplementary figures and images for: How laws affect the perception of norms: Empirical evidence from the lockdown
Source: PLoS One. 2021 Sep 24;16(9):e0256624. doi: 10.1371/journal.pone.0256624 (PMC8462721; doi:10.1371/journal.pone.0256624)

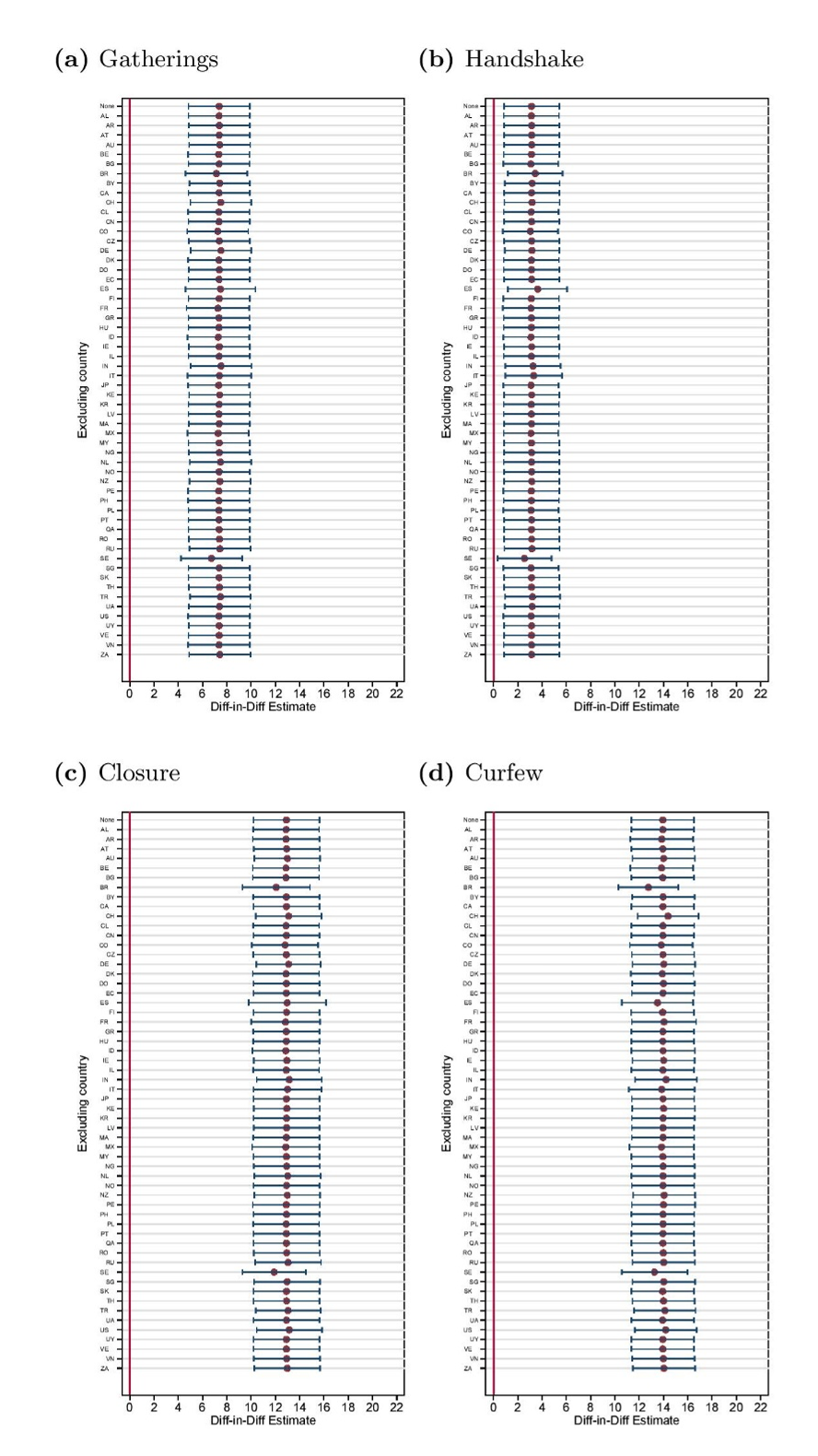

Supplement: S1 Fig — The figure reports the point estimates along with 95% confidence intervals resulting from the difference-in-difference estimation in (1) performed on control groups resulting from the step deletion of each country one after the other. The countries iso-code are defined in S1 Table. (TIF) [file pone.0256624.s004.tif]

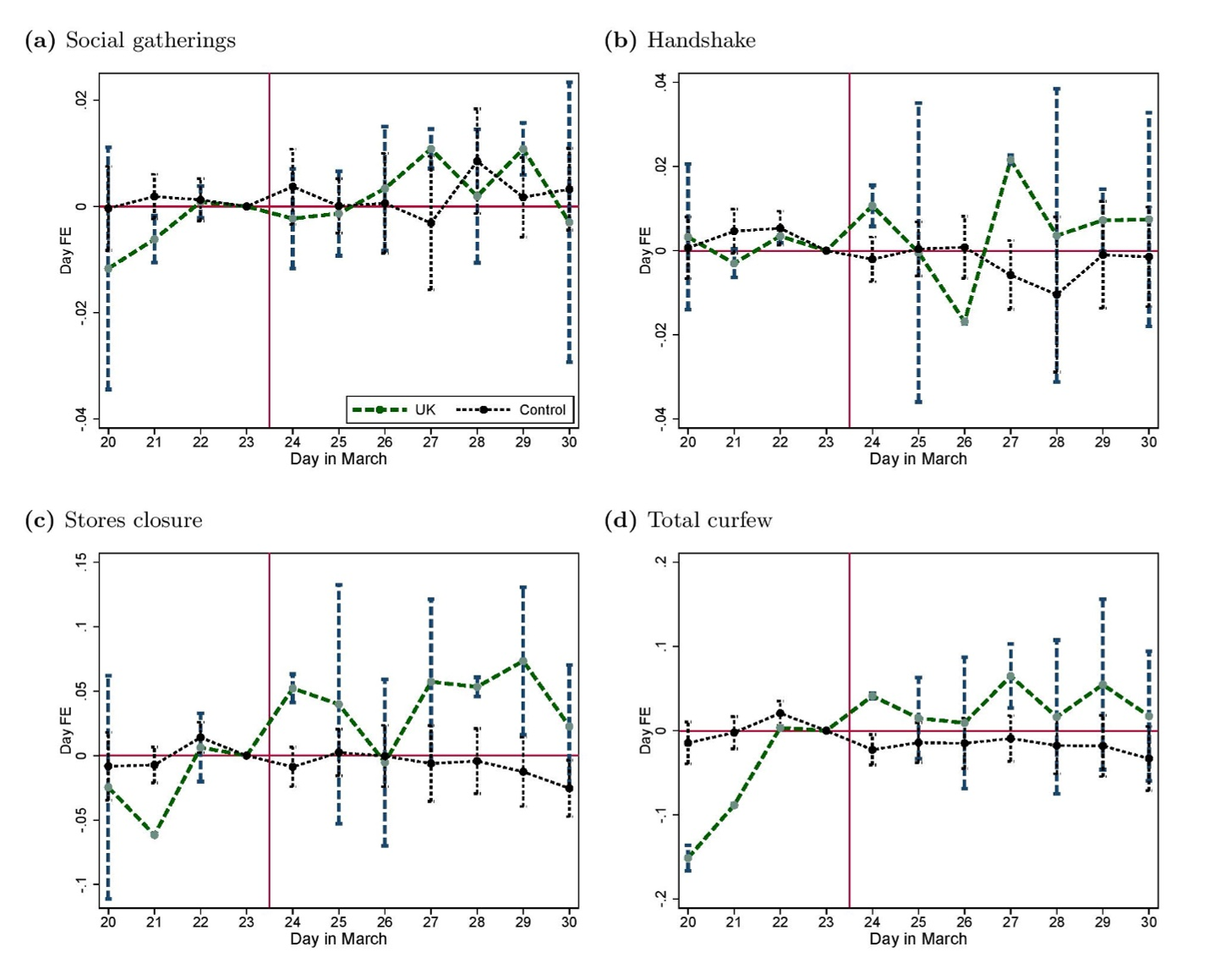

Supplement: S2 Fig — For each of the four policy measures, the figure reports the day-fixed effects from individual personal norms in the UK and in control group countries, controlling for country-, age-, gender-, education-, and income-fixed effects, as as well as a measure of household composition. The results from difference-in-difference estimates of the effect of March 23 announcement on individual personal norms, available from the authors upon request, confirm that the announcement has a non-significant effect regarding both handshaking and social gatherings, and a small but statistically significant effect regarding stores closure and a total curfew. (TIF) [file pone.0256624.s005.tif]
