# Supplementary material for: How laws affect the perception of norms: Empirical evidence from the lockdown
Source: PLoS One. 2021 Sep 24;16(9):e0256624. doi: 10.1371/journal.pone.0256624 (PMC8462721; doi:10.1371/journal.pone.0256624)
Supplement: S1 Table — Table reporting the distribution of respondents across countries. (PDF) [file pone.0256624.s006.pdf]

	iso-code	N	%
Brazil	BR	11,230	11.27
United Kingdom	GB	11,151	11.19
United States	US	11,060	11.10
**Germany**	**DE**	82,77	8.31
**Sweden**	**SE**	57,46	5.77
**Switzerland**	**CH**	4,152	4.17
Russia	RU	3,364	3.38
Mexico	MX	3,240	3.25
Turkey	TR	2,784	2.79
Canada	CA	2,709	2.72
**France**	**FR**	2,632	2.64
Belarus	BY	2,621	2.63
Spain	ES	2,211	2.22
Italy	IT	1,794	1.80
Colombia	CO	1,633	1.64
Indonesia	ID	1,541	1.55
Ukraine	UA	1,440	1.45
**Netherlands**	**NL**	1,346	1.35
**Austria**	**AT**	1,042	1.05
Peru	PE	1,015	1.02
India	IN	935	0.94
Qatar	QA	860	0.86
Argentina	AR	858	0.86
Australia	AU	852	0.86
Romania	RO	791	0.79
**Finland**	**FI**	756	0.76
Philippines	PH	731	0.73
**Ireland**	**IE**	695	0.70
Venezuela	VE	655	0.66

	iso-code	N	%
Vietnam	VN	634	0.64
Slovakia	SK	609	0.61
**Latvia**	**LV**	601	0.60
**Belgium**	**BE**	551	0.55
Dominican Republic	DO	546	0.55
Portugal	PT	542	0.54
Chile	CL	522	0.52
Malaysia	MY	512	0.51
**Denmark**	**DK**	504	0.51
Albania	AL	468	0.47
South Africa	ZA	468	0.47
Israel	IL	403	0.40
Singapore	SG	395	0.40
Poland	PL	377	0.38
Morocco	MA	351	0.35
Kenya	KE	340	0.34
China	CN	333	0.33
New Zealand	NZ	330	0.33
Bulgaria	BG	313	0.31
Greece	GR	310	0.31
Thailand	TH	303	0.30
Ecuador	EC	299	0.30
**Norway**	**NO**	292	0.29
South Korea	KR	275	0.28
Japan	JP	274	0.28
Czechia	CZ	256	0.26
Uruguay	UY	240	0.24
Hungary	HU	232	0.23
Nigeria	NG	212	0.21
Total		99,613	100.00

Note. Countries in bold letters are part of the Northern and Western European countries sub-sample.
