# Supplementary material for: How laws affect the perception of norms: Empirical evidence from the lockdown
Source: PLoS One. 2021 Sep 24;16(9):e0256624. doi: 10.1371/journal.pone.0256624 (PMC8462721; doi:10.1371/journal.pone.0256624)
Supplement: S2 Table — Table providing a comparison of the descriptive statistics observed in the UK and in other countries. (PDF) [file pone.0256624.s007.pdf]

	**Control**	**UK**	**Overall**
**Women**	56.6%	50.4%	55.9%
**Years of Education**	16.20<br>(4.815)	17.26<br>(3.713)	16.33<br>(4.711)
**Age**	38.22<br>(12.84)	43.54<br>(12.89)	38.85<br>(12.96)
**Single**	45.1%	33.0%	43.7%
**Number of Household Members**	2.863<br>(1.591)	2.664<br>(1.356)	2.839%<br>(1.566)
**First income quintile**	8.60%	10.68%	10.43%
**Second income quintile**	6.51%	6.66%	6.64%
**Third income quintile**	4.90%	7.18%	6.91%
**Fourth income quintile**	12.13%	12.56%	12.51%
**Fourth income quintile**	67.86%	62.92%	63.50%
**Confirmed COV-19 cases p.c.**	0.206<br>(0.290)	0.0978<br>(0.0409)	0.193%<br>(0.275)
**Lag confirmed COV-19 cases p.c.**	0.0259<br>(0.0339)	0.0141<br>(0.00676)	0.0245%<br>(0.0322)
**Confirmed COV-19 deaths p.c.**	0.00591<br>(0.0169)	0.00553<br>(0.00410)	0.00587%<br>(0.0159)
**Lag Confirmed COV-19 cases p.c.**	0.00106<br>(0.00244)	0.000985<br>(0.000863)	0.00105%<br>(0.00231)

Note. This table presents covariate means for control group countries, the UK, and the overall sample. The notation p.c. corresponds to per country. Standard deviation is reported in parentheses.
