# Supplementary material for: How laws affect the perception of norms: Empirical evidence from the lockdown
Source: PLoS One. 2021 Sep 24;16(9):e0256624. doi: 10.1371/journal.pone.0256624 (PMC8462721; doi:10.1371/journal.pone.0256624)
Supplement: S3 Table — This Table reports the results of the Difference-in-Difference estimation (Post × UK variable) of the March 23 lockdown decision in the UK on mobility data collected from Google’s publicly available community mobility reports. Each outcome variable is the change in spending time at a given place relative to the median value of the same weekday in the January 3-February 6 period. The specification controls for COVID-19 statistics (lagged and current confirmed cases and deaths) as well as a non-linear effect of the group and Post variable through day- and country fixed-effects. (PDF) [file pone.0256624.s008.pdf]

	**Retail /recreation**	**Pharmacy /Grocery**	**Parks**	**Transit**
**A. Full sample** ($N = 960$; 124 Clusters)				
Post × UK	-25.642***<br>(1.024)	-21.746***<br>(1.167)	-29.655***<br>(2.007)	-16.521***<br>(0.987)
Lag confirmed COV-19 cases p.c.	14.350<br>(10.109)	49.672<br>(31.207)	3.192<br>(25.382)	16.562*<br>(8.224)
Lag confirmed COV-19 deaths p.c.	114.559<br>(221.077)	-39.607<br>(331.812)	-1378.007<br>(827.303)	-85.451<br>(175.091)
Confirmed COV-19 cases p.c.	9.915*<br>(3.825)	8.681*<br>(3.393)	5.391<br>(3.855)	9.175*<br>(4.151)
Confirmed COV-19 deaths p.c.	32.310<br>(49.066)	-24.885<br>(46.384)	61.112<br>(58.136)	36.770<br>(45.195)
**B. Western and Northern Europe** ($N = 167$; 16 Clusters)				
Post × UK	-30.497***<br>(1.379)	-26.002***<br>(1.470)	-29.849***<br>(3.774)	-20.610***<br>(1.064)
Lag confirmed COV-19 cases p.c.	4.168<br>(6.110)	24.933<br>(34.540)	-49.647<br>(28.724)	2.981<br>(5.409)
Lag confirmed COV-19 deaths p.c.	-703.338<br>(521.176)	-594.192<br>(457.703)	-3024.148<br>(1572.284)	-627.219<br>(332.433)
Confirmed COV-19 cases p.c.	2.174<br>(1.618)	2.860<br>(2.114)	7.658*<br>(3.262)	1.882<br>(1.693)
Confirmed COV-19 deaths p.c.	176.052<br>(115.063)	-60.292<br>(98.899)	234.398<br>(324.278)	75.669<br>(91.025)

Note. The notation p.c. corresponds to per country. Standard errors are reported in parentheses and clustered at the country level. *Significance levels*: *5%, **1%, ***0.1%.
