# Supplementary material for: How laws affect the perception of norms: Empirical evidence from the lockdown
Source: PLoS One. 2021 Sep 24;16(9):e0256624. doi: 10.1371/journal.pone.0256624 (PMC8462721; doi:10.1371/journal.pone.0256624)
Supplement: S4 Table — (PDF) [file pone.0256624.s009.pdf]

	Leaves Home	Friends	Bored	Tired	Work	Freedom
**Perceived social norms (Curfew)**	-0.058	-0.056***	-0.023***	-0.048***	-0.048***	-0.008
	(0.037)	(0.014)	(0.005)	(0.011)	(0.011)	(0.006)
**Personal norms (Curfew)**	-0.167***	-0.059***	-0.054***	-0.101***	-0.101***	-0.034***
	(0.015)	(0.007)	(0.006)	(0.005)	(0.005)	(0.005)
**Constant**	0.743***	0.118***	0.089***	0.184***	0.184***	0.048***
	(0.032)	(0.015)	(0.008)	(0.011)	(0.011)	(0.008)

Note. The survey contains information on whether respondents intend to leave their home in the course of the next five days and for what motives. This table reports the results from an OLS regression of these mobility variables on the personal norm about a curfew and the perceived social norm to support a curfew. We build the variables from two series of the survey questions: the first asks "*Do you need to leave your home in the next 5 days?*" [yes = 1; no =0]. The second is phrased "*What are the reasons for you to leave your home (check all that apply)?*"; among all possible answers, we focus on: [Friends] ("*Meeting friends or relatives*"); [Tired] ("*Getting tired of being inside of the house*"); [Bored] ("*Getting bored*"); [Work] ("*Going to work*"); [Freedom] ("*Exercising my freedom*"). The results support the existence a relation between mobility behavior and both the personal norms and the perception of social norms: respondents who believe that others support a curfew are less likely to state that they plan to leave their home in the coming days. Standard errors are reported in parentheses and clustered on the country-gender level ($N = 99,613$; 170 clusters). The perceived social norm explanatory variable is the original variable from the survey but divided by 100 to make it comparable with the social norm. *Significance levels*: *5%, **1%, ***0.1%.
