# Supplementary material for: How laws affect the perception of norms: Empirical evidence from the lockdown
Source: PLoS One. 2021 Sep 24;16(9):e0256624. doi: 10.1371/journal.pone.0256624 (PMC8462721; doi:10.1371/journal.pone.0256624)
Supplement: S5 Table — (PDF) [file pone.0256624.s010.pdf]

	Gatherings	Handshake	Stores	Curfew
**A. Full sample** ($N = 94,544$; 155 clusters)				
Post x UK	7.370***	3.133**	12.907***	13.950***
	(1.287)	(1.158)	(1.386)	(1.311)
Lagged confirmed COV-19 cases p.c.	-0.090	-7.487	-4.801	-9.290
	(13.873)	(10.020)	(14.224)	(14.560)
Lagged confirmed COV-19 deaths p.c.	-253.572	-24.141	216.299	298.979
	(261.672)	(262.240)	(275.434)	(361.588)
Confirmed COV-19 cases p.c.	-7.885***	-7.108***	-6.045	-8.313
	(1.676)	(1.233)	(3.455)	(4.608)
Confirmed COV-19 deaths p.c.	59.200***	24.718	-30.181	-31.590
	(15.496)	(20.375)	(25.644)	(28.736)
Household size	0.189***	0.109	0.292***	0.481***
	(0.055)	(0.063)	(0.059)	(0.064)
Constant	68.304***	76.253***	58.690***	48.829***
	(0.346)	(0.316)	(0.654)	(0.855)
**B. Western and Northern Europe** ($N = 37,745$; 38 clusters)				
Post x UK	7.825***	4.262***	14.284***	14.405***
	(1.429)	(1.185)	(1.913)	(1.764)
Lagged confirmed COV-19 cases p.c.	-1.267	-4.035	-17.841	-34.271*
	(18.043)	(11.962)	(16.923)	(16.785)
Lagged confirmed COV-19 deaths p.c.	-362.520	-281.885	455.944	1179.429
	(490.437)	(446.046)	(421.836)	(587.650)
Confirmed COV-19 cases p.c.	-6.500***	-3.496*	-0.432	-7.228
	(1.608)	(1.454)	(5.654)	(5.974)
Confirmed COV-19 deaths p.c.	117.739	42.186	18.662	-99.899
	(62.331)	(53.258)	(68.630)	(108.627)
Household size	0.145**	0.209***	0.189**	0.468***
	(0.046)	(0.051)	(0.056)	(0.084)
Constant	72.534***	83.664***	61.210***	51.851***
	(0.859)	(0.599)	(2.076)	(2.243)

Note. Standard errors are reported in parentheses and clustered at the country-gender level. *Significance levels*: *5%, **1%, ***0.1%.
