# Supplementary material for: How laws affect the perception of norms: Empirical evidence from the lockdown
Source: PLoS One. 2021 Sep 24;16(9):e0256624. doi: 10.1371/journal.pone.0256624 (PMC8462721; doi:10.1371/journal.pone.0256624)
Supplement: S6 Table — (PDF) [file pone.0256624.s011.pdf]

| | **Gatherings** | **Handshake** | **Stores** | **Curfew** |
|---|---|---|---|---|
| **A. Full sample** ($N = 91,182$; 137 clusters) | | | | |
| Post x UK | -6.182*** | -0.278 | -6.037*** | -3.910* |
| | (1.518) | (1.499) | (1.649) | (1.630) |
| Lagged confirmed COV-19 cases p.c. | 7.022 | 13.025 | 16.393 | 51.882* |
| | (14.491) | (10.938) | (14.992) | (22.504) |
| Lagged confirmed COV-19 deaths p.c. | 344.059 | 115.689 | 115.465 | -595.692 |
| | (333.648) | (248.481) | (355.525) | (303.982) |
| Confirmed COV-19 cases p.c. | 7.849*** | 7.387 | -0.077 | -1.014 |
| | (1.923) | (4.296) | (4.429) | (6.441) |
| Confirmed COV-19 deaths p.c. | -87.380*** | 28.834 | -75.645 | 20.271 |
| | (18.264) | (26.188) | (49.525) | (45.952) |
| Household size | -0.175** | -0.127 | -0.252*** | -0.453*** |
| | (0.057) | (0.064) | (0.060) | (0.069) |
| **B. Western and Northern Europe** ($N = 37,745$; 38 clusters) | | | | |
| Post x UK | -7.622*** | -1.699 | -6.773* | -4.269* |
| | (2.020) | (1.153) | (2.979) | (1.958) |
| Lagged confirmed COV-19 cases p.c. | 2.137 | -6.573 | -7.529 | 45.302 |
| | (17.054) | (14.996) | (20.810) | (36.197) |
| Lagged confirmed COV-19 deaths p.c. | 325.700 | 280.957 | -709.197 | 512.372 |
| | (578.627) | (450.184) | (639.362) | (491.423) |
| Confirmed COV-19 cases p.c. | -0.615 | 2.965 | -10.248 | -8.838 |
| | (3.537) | (2.085) | (8.472) | (6.000) |
| Confirmed COV-19 deaths p.c. | -212.282* | 76.330 | -84.867 | -242.935* |
| | (80.470) | (56.498) | (160.257) | (109.129) |
| Household size | -0.145** | -0.211*** | -0.186** | -0.457*** |
| | (0.046) | (0.051) | (0.056) | (0.083) |

**Note.** Standard errors are reported in parentheses and clustered at the country-gender level. *Significance levels*: *5%, **1%, ***0.1%.
