# Supplementary material for: How laws affect the perception of norms: Empirical evidence from the lockdown
Source: PLoS One. 2021 Sep 24;16(9):e0256624. doi: 10.1371/journal.pone.0256624 (PMC8462721; doi:10.1371/journal.pone.0256624)
Supplement: S7 Table — This table provides the DiD estimate in (1) using weights. The weights provide representativity on the country level by age, income quintile, and education. (PDF) [file pone.0256624.s012.pdf]

Table 1. DiD estimates of the effect of the UK lockdown using weighted data

	**Gatherings**	**Handshake**	**Stores**	**Curfew**	*N*	Clusters
		**D. Weighted data**				
Perceived norm	7.079***	3.397*	13.313***	16.698***	90,396	94
	(1.473)	(1.343)	(2.066)	(1.679)		
Western and Northern Europe	7.447***	3.040	15.487***	18.092***	37,357	26
	(1.756)	(1.837)	(2.548)	(2.224)		

Note. Standard errors are reported in parentheses and clustered at the country-gender level. *Significance levels*: *5%, **1%, ***0.1%.
