# Supplementary material for: How laws affect the perception of norms: Empirical evidence from the lockdown
Source: PLoS One. 2021 Sep 24;16(9):e0256624. doi: 10.1371/journal.pone.0256624 (PMC8462721; doi:10.1371/journal.pone.0256624)
Supplement: S8 Table — (PDF) [file pone.0256624.s013.pdf]

Table 1. Heterogeneity of the DiD estimate according to individual covariates

	**Gatherings**	**Handshake**	**Stores**	**Curfew**
**A: Gender** (the direct effect is part of the model FE)				
Post x UK	13.228***	7.957***	17.567***	18.120***
	(0.816)	(0.547)	(1.000)	(1.047)
Post x UK x Female	-3.599***	-2.964***	-2.863***	-2.562***
	(0.351)	(0.102)	(0.217)	(0.126)
**B. Income bracket** (the direct effect is part of the model FE)				
Post x UK	6.142***	3.193***	10.268***	13.022***
	(0.656)	(0.674)	(1.537)	(1.856)
Post x UK x Income quintile	0.295	-0.015	0.633	0.223
	(0.369)	(0.315)	(0.561)	(0.633)
**C. Years of education**				
Post x UK	9.449***	5.049***	15.936***	12.795***
	(1.362)	(1.160)	(1.027)	(1.978)
Years of education	-0.023	0.004	-0.125***	-0.298***
	(0.018)	(0.014)	(0.026)	(0.034)
Post x UK x Years of education	-0.127***	-0.115***	-0.179**	0.050
	(0.033)	(0.021)	(0.055)	(0.049)
**D. Household size**				
Post x UK	6.503***	2.870**	12.578***	12.383***
	(0.833)	(1.055)	(1.332)	(1.471)
Household size	0.184**	0.108	0.290***	0.472***
	(0.056)	(0.064)	(0.060)	(0.064)
Post x UK x Household size	0.329	0.100	0.125*	0.595***
	(0.221)	(0.084)	(0.063)	(0.121)

Note. Standard errors are reported in parentheses and clustered on the country-gender level ($N = 94,544$; 155 clusters). *Significance levels*: *5%, **1%, ***0.1%.
