# Supplementary material for: How laws affect the perception of norms: Empirical evidence from the lockdown
Source: PLoS One. 2021 Sep 24;16(9):e0256624. doi: 10.1371/journal.pone.0256624 (PMC8462721; doi:10.1371/journal.pone.0256624)
Supplement: S9 Table — (PDF) [file pone.0256624.s014.pdf]

	Gatherings	Handshake	Stores	Curfew
**A. Misestimation of Covid cases**				
Post x UK	7.260***	3.059*	12.649***	13.544***
	(1.194)	(1.177)	(1.376)	(1.303)
Mistake	-0.002**	-0.003***	-0.003**	-0.001
	(0.001)	(0.001)	(0.001)	(0.001)
Post x UK x Mistake	0.011	0.008*	0.025***	0.038***
	(0.010)	(0.003)	(0.004)	(0.004)
**B. Subjective effectiveness of containment measures**				
Post x UK	8.441***	4.780*	17.471***	14.315***
	(1.250)	(2.067)	(1.403)	(2.332)
Subjective Effectiveness	0.581***	0.516***	0.944***	1.153***
	(0.147)	(0.151)	(0.181)	(0.226)
Post x UK x Subjective Effectiveness	-0.272	-0.409	-1.124***	-0.114
	(0.507)	(0.727)	(0.283)	(0.287)
**C. Subjective trust in government**				
Post x UK	6.260***	3.855**	10.357***	12.423***
	(0.719)	(1.210)	(1.048)	(1.028)
Trust in Government	2.696***	2.454***	2.387***	2.064***
	(0.098)	(0.124)	(0.166)	(0.220)
Post x UK x Trust in Government	0.028	-0.538	0.535	0.242
	(0.497)	(0.648)	(0.570)	(0.450)
**D. Government was truthful**				
Post x UK	7.705***	5.230***	11.710***	11.366***
	(0.702)	(1.321)	(0.861)	(1.212)
Government was truthful	2.450***	2.267***	2.053***	1.595***
	(0.104)	(0.122)	(0.158)	(0.206)
Post x UK x Government was truthful	-0.290	-0.796	0.192	0.637**
	(0.432)	(0.650)	(0.334)	(0.226)

Note. Standard errors are reported in parentheses and clustered on the country-gender level ($N = 94,544$; 155 clusters). *Significance levels*: *5%, **1%, ***0.1%.
